# The Epigenetics of Emerging and Nonmodel Organisms

**DOI:** 10.1155/2012/491204

**Published:** 2012-11-28

**Authors:** Vett K. Lloyd, Jennifer A. Brisson, Kathleen A. Fitzpatrick, Lori A. McEachern, Eveline C. Verhulst

**Affiliations:** ^1^Department of Biology, Mount Allison University, 63B York Street, Sackville, NB, Canada E4L 1G7; ^2^School of Biological Sciences, University of Nebraska, Lincoln, NE 68588, USA; ^3^Department of Biological Sciences, Simon Fraser University, 8888 University Drive, Burnaby, BC, Canada V5A 1S6; ^4^Department of Physiology & Biophysics, Dalhousie University, 5850 College Street, Halifax, NS, Canada B3H 4R2; ^5^Evolutionary Genetics Group, Department of Life Sciences, University of Groningen, P.O. Box 11103, 9700 CC Groningen, The Netherlands

Genetic model organisms have gifted researchers with a breathtakingly detailed understanding of the most intimate aspects of their genomes, cells, and development. And yet there is a problem—model organisms have been selected because they have simple life histories and happily inhabit laboratories. In short, they make a virtue of being boring. But the diversity of the natural world is not fully captured by yeast, flies, or mice. To truly appreciate the variety of biological mechanisms underlying this remarkable diversity, one must study the often inconvenient but fascinating non-model organism. Experimental and descriptive approaches in nonmodel organisms have become more tractable with reduced genome-sequencing costs and the transferability of techniques and tools developed in model organisms, elevating some of them from non-model to emerging model organism status.

One area of biology into which non-model organisms promise to provide significant insight is the area of epigenetics. Epigenetics focuses on how internal and external environments interact with the genome to produce the phenotype, and non-model organisms arguably present a larger range of phenotypes than model organisms. In this issue, we present recent research into the epigenetics of non- and emerging-model organisms. The papers in this series highlight several common themes: experimental approaches to studying epigenetics in non-model organisms, epigenetics as a mediator of environmental changes in morphology and development, and epigenetic contributions to individual and population diversity.

**How to Study Epigenetics in Non- and Emerging-Model Organisms**

Despite the ecological and evolutionary importance of non-model organisms, an obvious disadvantage is the absence of genetic and epigenetic tools available for these organisms. This issue is addressed by W. A. MacDonald, who examines the question of whether one of the best-studied aspects of epigenetics, genomic imprinting, is evolutionarily conserved. His conclusion that the basic epigenetic mechanisms, if not the target genes, are conserved, allows the extrapolation of findings from model organisms to non-model organisms. This approach is taken by studies on polychaetes (G. Gibson et al.) and *Daphnia* (N. F. Robichaud et al.) in this special issue. L. A. McEachern further explores the potential of transgenic epigenetic studies in non-model organism research. This underutilized but powerful and sophisticated approach to studying epigenetics involves transferring a potential epigenetic control sequence from one organism to another for detailed molecular analysis. On a practical level, G. Prantera and S. Bongiorni examine new experimental approaches used to dissect one of the first epigenetic processes described, chromosome imprinting in mealybugs, and K. R. Shorter et al. describe the manifold resources for the study of the deer mouse *Peromyscus*, by the *Peromyscus* genome center.

**Epigenetics as a Mediator of Environmentally Driven Changes in Morphology and Development**

A mouse looks like a mouse—one tail, four feet, two ears, one nose, and so forth. A stressed mouse still looks like a mouse. The same is true for fruit flies, nematodes, and a great many other animals. However, there are some animals, which in response to environmental cues, alter their morphology so dramatically that they would not be recognized as the same species as their unstressed, and sometimes genotypically identical, counterparts.

When exposed to predators, various *Daphnia* species can grow cuticular elaborations that would be the envy of any punk rocker: long pointy helmets, tail spikes, neck teeth, and so forth. In some cases, these can double the size of the animal, making them unpalatable to the predator. Similarly, environmental cues can change the sex of an individual, turning what would normally be a parthenogenetically reproducing female into a male, thereby allowing sexual reproduction. Morphological changes are also seen in aphids; under stress, the normally boxy-bodied sap-sucking machine can produce offspring that develop into a svelte winged form with a much smaller streamlined body with entirely different musculature. In this special issue, K. D. M. Harris et al. and D. G. Srinivasan and J. A. Brisson review what has been explored with respect to the epigenetic basis of these dramatic morphological differences in *Daphnia* and aphids, respectively.

Epigenetically mediated morphological differences are also observed in the genus *Onthophagus*, in which male beetles may grow large horns or not, depending on development time and consequent body size, and also display a corresponding plasticity in mating behaviour. Array experiments have identified candidate genes differentially expressed in the two “morphs”, and in this issue, S. Valena and A. P. Moczek discuss how differential DNA methylation may be involved in these expression changes. Similarly, some polycheate worms can alter their development to produce either small planktonic larvae optimized for dispersal or large yolk-filled larvae, which tend to settle near the parents. G. Gibson et al. provide the first information on the epigenetic basis of these polyphenisms. This theme is continued in the review of epigenetics in social insects, in which S. A. Weiner and A. L. Toth describe some interesting connections between differential DNA methylation and caste polyphenisms in social insects.

**Epigenetic Contributions to Individual and Population Diversity**

Epigenetic states can be inherited and thus can contribute to the evolutionary process. However, the role of epigenetic diversity in promoting selectable phenotypic diversity at the population level has not yet been well studied in animals. Here, A. W. Schrey et al. use methylation-sensitive-amplified fragment length polymorphism markers to measure epigenetic variation in introduced populations of house sparrows. With these results the authors hypothesize that this epigenetic variation may play a role in maintaining phenotypic diversity following the reduced genetic diversity that occurs with a genetic bottleneck. Using similar methods, R. Massicotte and B. Angers investigate the flexibility of the phenotype using the static genotype of a clonally reproducing fish. They report on different epigenetic modifications under different environmental conditions such as water pH. These two studies demonstrate that epigenetic modifications in natural populations and their relationship to the phenotype is a rich area for further exploration.

*Vett K. Lloyd* *Vett K. Lloyd* *Jennifer A. Brisson* *Jennifer A. Brisson* *Kathleen A. Fitzpatrick* *Kathleen A. Fitzpatrick* *Lori A. McEachern* *Lori A. McEachern* *Eveline C. Verhulst* *Eveline C. Verhulst*

